# Cognitive Function Recovery Pattern in Adult Patients With Severe Anti-N-Methyl-D-Aspartate Receptor Encephalitis: A Longitudinal Study

**DOI:** 10.3389/fneur.2018.00675

**Published:** 2018-08-20

**Authors:** Zhongqin Chen, Dengchang Wu, Kang Wang, Benyan Luo

**Affiliations:** Department of Neurology, The First Affiliated Hospital, Zhejiang University School of Medicine, Hangzhou, China

**Keywords:** anti-NMDAR encephalitis, adult patients, cognition, episodic memory, executive control

## Abstract

**Objective:** To observe the dynamic characteristics of cognitive function following early application of immunotherapy in adult patients with severe anti N-methyl D-aspartate receptor (anti-NMDAR) encephalitis.

**Methods:** Serial neuropsychological assessments were performed at three sequential time points in five adult patients with severe anti-NMDAR encephalitis following early-initiated immunotherapy. The three sequential points were 1–2, 6, and 11–12 months after treatment. Five normal subjects without psychological or neurological diseases were assessed as a control group.

**Results:** Following early-initiated immunotherapy, all five patients demonstrated a gradual improvement of overall cognitive function over the 1-year follow-up period. All patients had suffered from a comprehensive cognitive function disorder from the early stages of the illness. Six months after the immunotherapy, the treatment group showed no significant differences in verbal episodic memory function compared with the control group. One year after the immunotherapy, non-verbal episodic memory function in the treatment group had normalized. The results of other tests related to frontoparietal cognitive function revealed damage of varying degrees during these three phases.

**Conclusion:** The results of this sequential observation study indicated a three-phase recovery pattern of cognitive function in adult patients with severe anti-NMDAR encephalitis following early initiated immunotherapy. These findings extend current understanding of the recovery mechanisms of cognitive function impairment in this disease.

## Introduction

Comprehensive cognitive function impairment is one of the major symptoms in the early stages of anti N-methyl D-aspartate receptor (anti-NMDAR) encephalitis (1). Although approximately 80% of patients receiving timely immunotherapy present with modified Rankin Scores (mRS) in the lower range (1), many patients are left with persistent cognitive deficits, predominantly in the domains of episodic memory, attention, and executive control, which can eventually become long-term consequences of the disease (2, 3).

Because cognitive function impairment impacts on the life and work of patients after the illness has resolved, clinicians are increasingly paying attention to cognitive function impairments in anti-NMDAR encephalitis (2–7). In a recent systematic review, McKeon et al. assessed neuropsychological characteristics in different recovery stages of 81 anti-NMDAR encephalitis cases collected from different studies (6). The study concluded that over 75% of the patients had cognitive deficits, and the rates of performance impairments on tests of executive functioning, episodic memory, and processing speed were unrelated to assessment timing (6). However, most attention has been paid to the characteristics of cognitive function impairment in the chronic stages of the disease, particularly regarding the assessment of verbal episodic memory and executive control. In contrast, few studies have focused on nonverbal episodic memory and the dynamic characteristics of the progress of cognitive function recovery.

Although one study reported the progress of cognitive function changes in anti-NMDAR encephalitis, the participants in the study ranged in age from 3–17 years old (8). Thus, little is known regarding the progression and patterns of cognitive function changes in adult patients with severe anti-NMDAR encephalitis following immunotherapy at the early stages of the illness. Thus, in the current study, we conducted long-term neuropsychological follow-up of verbal and nonverbal episodic memory and other frontoparietal functions in adult patients with severe anti-NMDAR encephalitis, to investigate the characteristics of the progression and recovery of cognitive function impairment in these patients. This study sought to provide clear neuropsychological evidence to extend our understanding of the development of cognitive function impairment and the mechanisms underlying its recovery.

## Materials and methods

### Subjects

The subjects of this study were five adult patients with severe anti-NMDAR encephalitis who were admitted to hospital from October 2016 to December 2017. All patients were diagnosed in accordance with anti-NMDAR encephalitis criteria (9). The mean age was 26 ± 3.54 years. Three of the subjects were female and two were male.

The diagnosis was based on typical clinical features together with the presence of the IgG antibody for NMDA receptors (9, 10)_._ None of the patients presented obvious lesions on routine cranial magnetic resonance imaging (MRI), previous psychological disorders or seizure, or genetic neurological illness. The patients' duration of education ranged from 12–16 years, and all patients were able to competently perform their jobs before illness, according to their parents or spouse. At the early stage (within 3 months following the appearance of the first symptom), all patients received first-line (high dose steroids and intravenous human immunoglobulin) and second-line (intravenous administration of rituximab and cyclophosphamide) immunotherapy, and did not receive any cognitive stimulation during follow-up time.

mRS values were measured at the acute stage before treatment, after initial immunotherapy, at 1–2 months (T1), 6 months (T2), and 11–12 months (T3), and were evaluated independently by two experienced neurologists. The highest mRS value of all the patients during the acute stage was five, so all patients were considered to have severe anti-NMDAR encephalitis. All patients were in a coma and required continuous attention and care at the acute stage.

Five healthy subjects without any history of psychological or neurological disorders were also enrolled in this study as healthy controls (HCs). The HCs were individually matched to patients with respect to sex, education level, and age. Clinical and demographic information of the patients and HCs are shown in Table 1.

**Table 1:** Clinical and demographic information of the patients and healthy controls.

							**mRS**					**Steroids**			**AEDs**			**Antidepressants or anti-anxiety meds**		
**Paticipant**	**Sex**	**Age**	**Education**	**NMDAR-IgG CSF at onset**	**Acute disease symptoms**	**Paraneoplastic syndrome**	**Before treatment**	**T1**	**T2**	**T3**	**Treatment**	**T1**	**T2**	**T3**	**T1**	**T2**	**T3**	**T1**	**T2**	**T3**
Patient_1	F	22	12	1:32	Loss of consciousness, movement disorder, seizures, behavioral and psychological symptoms	None	5	1	1	0	Steroids, IVIg, Cyclophosphamide	Methylprednisolone (8 mg/day)	None	None	Topamax (150 mg/day), Sodium valproate (600 mg/day)	Topamax(50 mg/day)	Topamax(50 mg/day)	None	None	None
Patient_2	F	24	16	1:32	Loss of consciousness, movement disorder, seizures, behavioral and psychological symptoms	None	5	2	1	0	Steroids, IVIg, Cyclophosphamide	Methylprednisolone (4 mg/day)	None	None	Sodium valproate (500 mg/day)	None	None	None	None	None
Patient_3	F	25	12	1:32	Loss of consciousness, movement disorder, seizures, behavioral and psychological symptoms	Teratoma	5	2	1	0	Steroids, IVIg, Rituximab, Cyclophosphamide, Surgical removal of teratoma	Methylprednisolone (8 mg/day)	Methylprednisolone (4 mg/day)	None	Levetiracetam (500 mg/day)	None	None	None	None	None
Patient_4	M	28	16	1:32	Loss of consciousness, movement disorder, seizures, behavioral and psychological symptoms	None	5	2	1	0	Steroids, IVIg, Cyclophosphamide	Methylprednisolone (8 mg/day)	None	None	Oxcarbazepine (600 mg/day), Topamax (50 mg/day)	None	None	None	None	None
Patient_5	M	31	12	1:32	Loss of consciousness, movement disorder, seizures, behavioral and psychological symptoms	None	5	1	1	0	IVIg, Cyclophosphamide	None	None	None	None	None	None	None	None	None
HCs_1	F	24	12																	
HCs_2	F	26	16																	
HCs_3	F	24	16																	
HCs_4	M	26	16																	
HCs_5	M	32	12																	

*CSF, cerebrospinal fluid; mRS, modified Rankin Scores; AEDs, antiepileptic drugs; IVIg, intravenous human immunoglobulin*.

The project was approved by the Ethical Committee of the First Affiliated Hospital, Zhejiang University School of Medicine. Written informed consent was obtained from the HCs, the legal guardians of the patients at T1 and T2, and the patients at T3.

### Neuropsychological assessment

A series of neuropsychological evaluations were performed on the patients and control subjects. Following the early immunotherapy, each patient received three neuropsychological assessments. These were undertaken after initial immunotherapy at 1–2 months (T1), 6 months (T2), and 11–12 months (T3). The mRS value was obtained at the same time. The timing for the first neuropsychological assessment was conducted when the mRS value was two or less, because patients at this level are more capable of cooperating in lengthy neuropsychological tests. At this time, patients' physical symptoms were mild, and they were not experiencing pain or weakness. The neuropsychological tests were performed in a special neuropsychological assessment room, and evaluations were conducted by an experienced neurologist after formal neuropsychological test training.

The tests employed validated Chinese versions of standard instruments, and evaluated several domains, as follows: (1) General neuropsychological tests: Mini-Mental State Examination (MMSE), Montreal Cognitive Assessment (MoCA), Self-Rating Anxiety Scale (SAS) and Self-rating Depression Scale (SDS). The MMSE and MoCA are widely-used standardized mental state examination tools. Both scales provide a simple and feasible method for obtaining a rapid reflection of patients' cognitive function. The SAS and SDS are used to assess emotional state; (2) Episodic memory: verbal episodic memory test (Chinese version of the Verbal Learning Test, CAVLT) and nonverbal episodic memory test (Aggie Figures Learning Test, AFLT) (11); (3) Working memory: digit span test (forward and backward); (4) Executive control: Stroop color and word test; (5) Semantic fluency test: fruit and vegetables, animals; (6) Information processing speed: symbol-digit modalities test (SDMT); (7) Visual-spatial capacity: block design test.

### Data analyses

SPSS 16.0 Statistical Software for Windows (SPSS Inc., Chicago, IL, USA) was used for the following statistical analyses. In one analysis, we compared the test results at the different time points between the HCs and the patients. Two-sample *t*-tests were used for the analysis of continuous data. Bonferroni correction was used to reduce the risk of type 1 errors. Hence, a stricter threshold of 0.017 (0.05/3) as the level of statistical significance, and 0.033 (0.1/3) as the level of marginal statistical significance, were used to explain the results of these two-sample *t*-tests. The other analysis was a comparison of the test results of the patients at the three different time points. For this the single factor repeated measurement analysis of variance was used. If Mauchly's test of sphericity was significant, Greenhouse-Geisser correction was used.

## Results

### Demographic characteristics of participants

As shown in Table 2, there were no significant differences between the treatment group and HCs in terms of gender, age and level of education.

**Table 2:** Demographic characteristics of the patients and healthy controls.

	**Patients (Mean ± SD)**	**HCs (Mean ± SD)**	***t***	***p***
Age (years)	26 ± 3.54	26.4 ± 3.29	0.185	0.858
Education (years)	13.6 ± 2.19	14.4 ± 2.19	0.577	0.58
Gender	3 female; 2 male	3 female; 2 male	/	/

*HCs, healthy controls, two-sample t-test*.

### General neuropsychological tests

As demonstrated in Table 3, there were no significant differences between patients and HCs at T1, T2, and T3 in SAS and SDS scores.

**Table 3:** The results of neuropsychological tests of the patients and healthy controls.

	**T1**		**T2**		**T3**	
**Tests**	***t***	***p***	***t***	***p***	***t***	***p***
Stroop test (color dot)	3.40	0.009	0.87	0.410	0.10	0.920
Stroop test (color word)	3.57	0.019^#^	1.30	0.230	−0.57	0.587
SDMT	−3.94	0.004	−3.94	0.004	−2.41	0.043
Digit span test (forward)	−7.30	0.000	−4.71	0.002	−2.83	0.022
Digit span test (backward)	−6.51	0.000	−3.58	0.007	−3.58	0.007
Semantic fluency test (vegetable and fruit)	−6.30	0.000	−0.06	0.554^#^	0.09	0.934
Semantic fluency test (animal)	−4.01	0.004	−1.55	0.159	−1.02	0.337
Block Design Test	−4.07	0.004	−0.24	0.057^#^	−1.35	0.214
CAVLT (immediate memory following interference)	−2.42	0.042	−0.09	0.415	0.28	0.784
CAVLT (delayed recall)	−2.86	0.021	−0.06	0.541	0.21	0.838
CAVLT (recognition)	−1.42	0.229^#^	−1.00	0.374^#^	/	/
AFLT (immediate memory following interference)	−3.94	0.013^#^	−2.56	0.033	−0.69	0.511
AFLT (delayed recall)	−3.73	0.018^#^	−2.36	0.071^#^	−0.65	0.535
AFLT (recognition)	−2.90	0.044^#^	−2.01	0.109^#^	−1.00	0.374^#^
MMSE	4.00	0.002^#^	−2.75	0.052^#^	−1.00	0.374^#^
MoCA	4.40	0.007^#^	−3.54	0.008	−2.13	0.066
SAS	0.92	0.385	0.13	0.901	1.00	0.346
SDS	0.23	0.822	0.06	0.958	0.64	0.543

CAVLT, Chinese auditory verbal learning test, AFLT, Aggie Figures Learning Test; SDMT, symbol-digit modalities test; SAS, self-rating anxiety scale; SDS, self-rating depression scale; MMSE, mini-mental state examination; MoCA, Montreal cognitive assessment. T1 = 1 to 2 months following initial immunotherapy; T2 = 6 months following initial immunotherapy; T3 = 11 to 12 months following; two-sample t-test;

# *variance inhomogeneity*.

**Figure 1:**
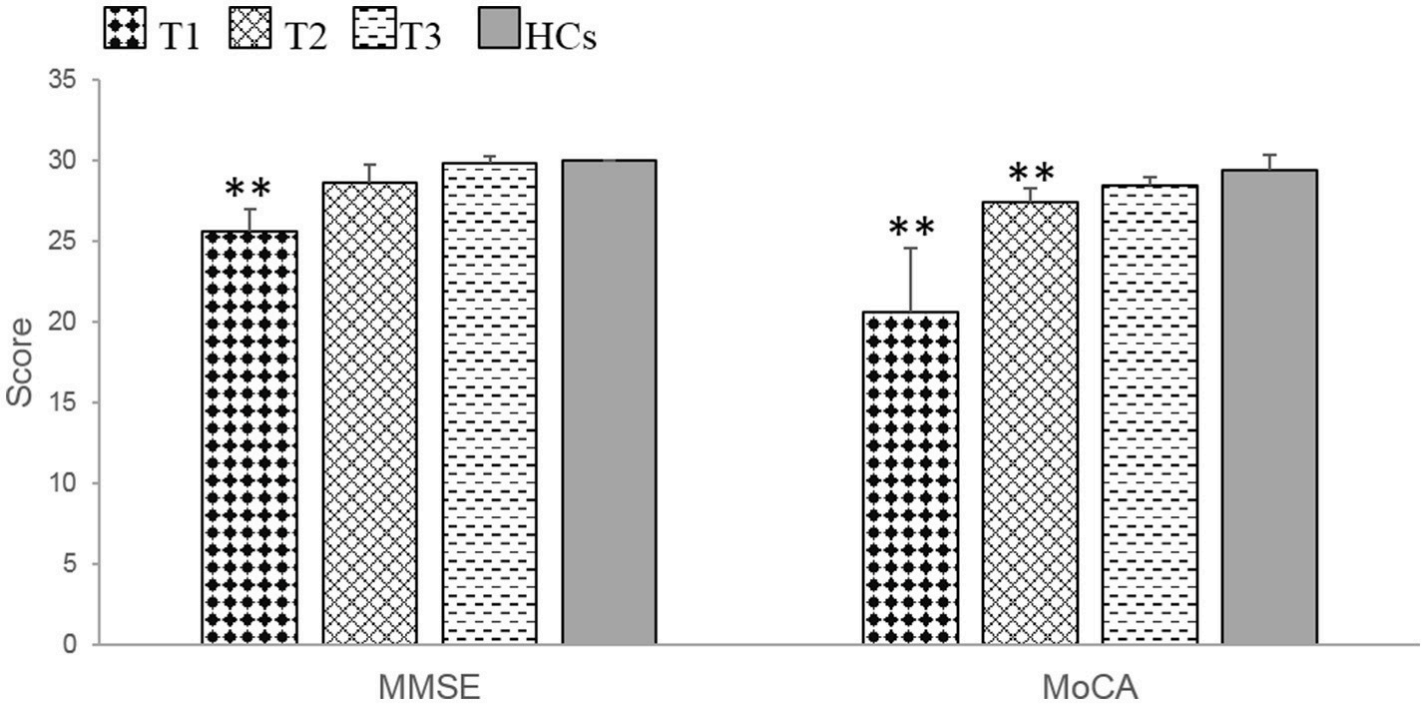
The patients with anti-NMDAR encephalitis showed significant general cognitive impairments at T1 and demonstrated obvious improvements during the recovery period. MMSE, mini-mental state examination. MoCA, Montreal cognitive assessment. T1 = 1 to 2 months following initial immunotherapy. T2 = 6 months following initial immunotherapy. T3 = 11 to 12 months following initial immunotherapy. The two-sample *t*-test between the patients and health controls (HCs) at three time points. **p < 0.05/3 (Bonferroni correction).

At T1, the patients exhibited significant impairments in MMSE (*t* = 4.00, *p* = 0.002) and MoCA (*t* = 4.40, *p* = 0.007) scores compared with HCs. At T2, the patients only demonstrated a significant impairment in the MoCA (*t* = −3.54, *p* = 0.008), whereas at T3, there were no significant differences between patients and HCs in MMSE and MoCA scores (Table 3, Figure 1).

During the recovery period, patients demonstrated significant cognitive improvements according to the MMSE (*F* = 23.80, *p* = 0.000) and MoCA (*F* = 15.71, *p* = 0.016) (Table 4, Figure 1). Further multiple comparisons revealed significant improvements in the results of these two tests at all three time points: MMSE (T1 vs. T2, *p* = 0.040; T1 vs. T3, *p* = 0.006) and MoCA (T1 vs. T3, *p* = 0.047) (Table 4).

**Table 4:** The results of neuropsychological tests at three time points of the patients.

	**Total**				
**Tests**	***F***	***p***	**T1 vs. T2**	**T1 vs. T3**	**T2 vs. T3**
Stroop test (color dot)	24.12	0.000	0.001	0.009	0.967
Stroop test (color word)	8.07	0.012	0.152	0.093	0.608
SDMT	5.45	0.032	0.339	0.206	0.269
Digit span test (forward)	21.00	0.001	0.009	0.010	1.000
Digit span test (backward)	6.32	0.023	0.155	0.155	1.000
Semantic fluency test (vegetable and fruit)	12.05	0.023^#^	0.093	0.065	0.105
Semantic fluency test (animal)	12.43	0.004	0.082	0.041	1.000
Block Design Test	13.38	0.003	0.275	0.017	0.081
CAVLT (immediate memory following interference)	6.10	0.025	0.052	0.171	0.99
CAVLT (delayed recall)	16.33	0.001	0.003	0.040	0.889
CAVLT (recognition)	2.10	0.221^#^	0.599	0.687	1.000
AFLT (immediate memory following interference)	9.62	0.007	0.207	0.046	0.344
AFLT (delayed recall)	7.32	0.016	0.376	0.061	0.358
AFLT (recognition)	6.94	0.018	0.155	0.170	0.567
MMSE	23.80	0.000	0.040	0.006	0.326
MoCA	15.71	0.016^#^	0.053	0.047	0.102

CAVLT, Chinese auditory verbal learning test; AFLT, Aggie Figures Learning Test; SDMT, symbol-digit modalities test; MMSE, mini-mental state examination; MoCA, Montreal cognitive assessment. T1 = 1 to 2 months following initial immunotherapy; T2 = 6 months following initial immunotherapy; T3 = 11 to 12 months following; repeated measurement analysis of variance;

# *Greenhouse-Geisser correction*.

### Neuropsychological tests of different cognitive domains

#### Episodic memory

**Figure 2:**
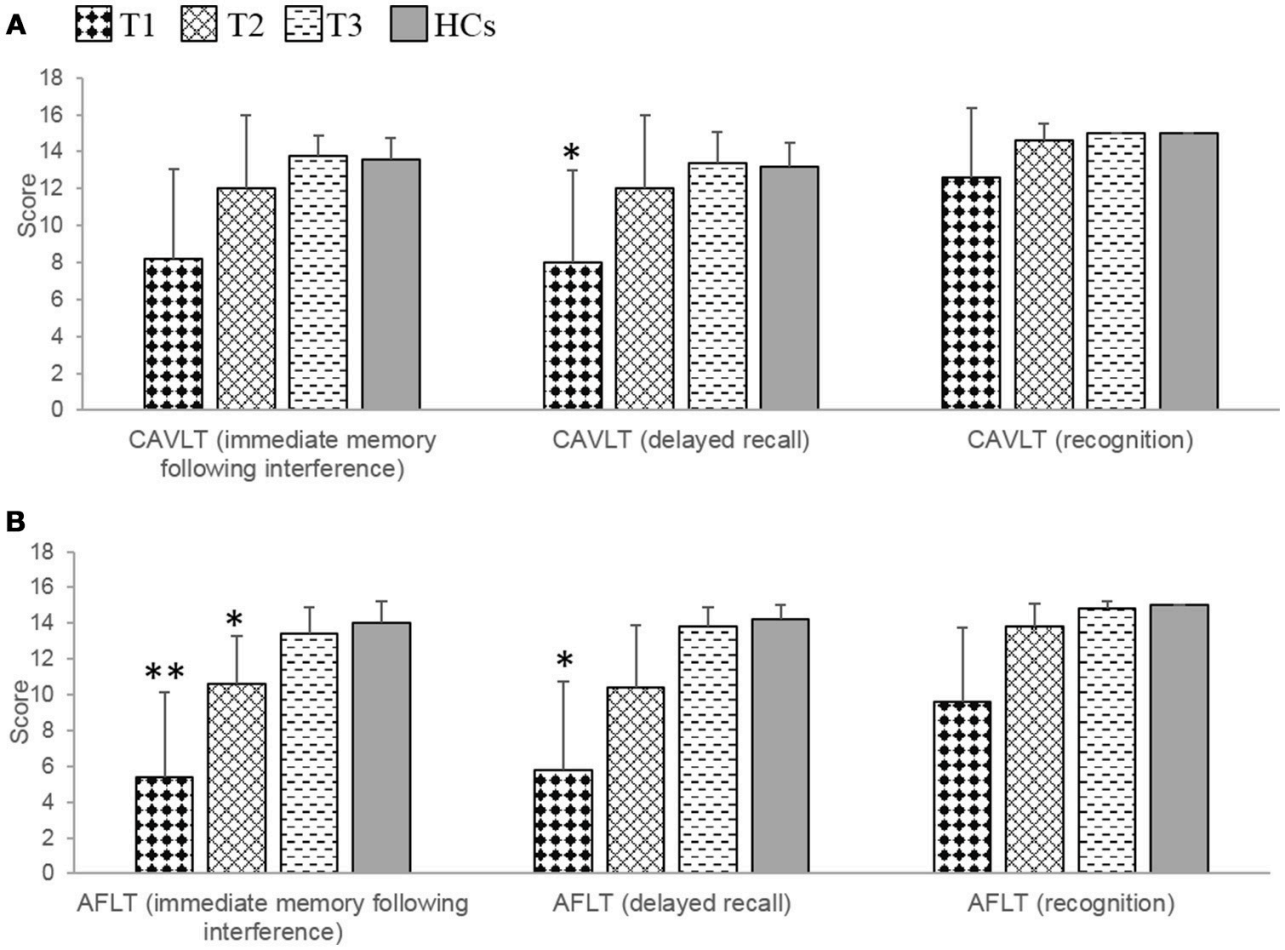
The results of episodic memory in the patients with anti-NMDAR encephalitis and health controls (HCs). **(A)** The patients showed marginally significant verbal episodic memory impairments at T1, and no obvious damage at T2 and T3 during the recovery period. **(B)** The patients showed significant or marginally significant non-verbal episodic memory impairments at T1 and T2, and no obvious damage at T3 during the recovery period. CAVLT, Chinese version of verbal learning test. AFLT, Aggie figures learning test. T1 = 1 to 2 months following initial immunotherapy. T2 = 6 months following initial immunotherapy. T3 = 11 to 12 months following initial immunotherapy. The two-sample *t*-test between the patients and health controls (HCs) at three time points. **p < 0.05/3; *p < 0.1/3 (Bonferroni correction).

At T1, significant or marginally significant impairments of verbal and nonverbal episodic memory were observed in patients compared with HCs, which was reflected mainly in the CAVLT (delayed recall, *t* = −2.86, *p* = 0.021) and AFLT (immediate memory following interference, *t* = −3.94, *p* = 0.013; delayed recall, *t* = −3.73, *p* = 0.018). At T2, there were no significant differences between patients and HCs in terms of CAVLT. However, compared with HCs, patients still presented with marginally significantly impairment in one component of the AFLT (recall following interference, *t* = −2.56, *p* = 0.033). At T3, further improvements were observed, and there were no significant differences between patients and HCs in AFLT (Table 3, Figures 2A,B).

During the recovery period, patients showed a significant improvement not only in verbal episodic memory (CAVLT: immediate memory following interference, *F* = 6.10, *p* = 0.025; delayed recall, *F* = 16.33, *p* = 0.001), but also in nonverbal episodic memory (AFLT: immediate memory following interference, *F* = 9.62, *p* = 0.007; delayed recall, *F* = 7.32, *p* = 0.016; recognition, *F* = 6.94, *p* = 0.018). Further multiple comparisons revealed significant improvements in the results of these two tests at all three time points: CAVLT (delayed recall, T1 vs. T2, *p* = 0.003; T1 vs. T3, *p* = 0.040), AFLT (immediate memory following interference, T1 vs. T3, *p* = 0.046) (Table 4).

### Working memory

**Figure 3:**
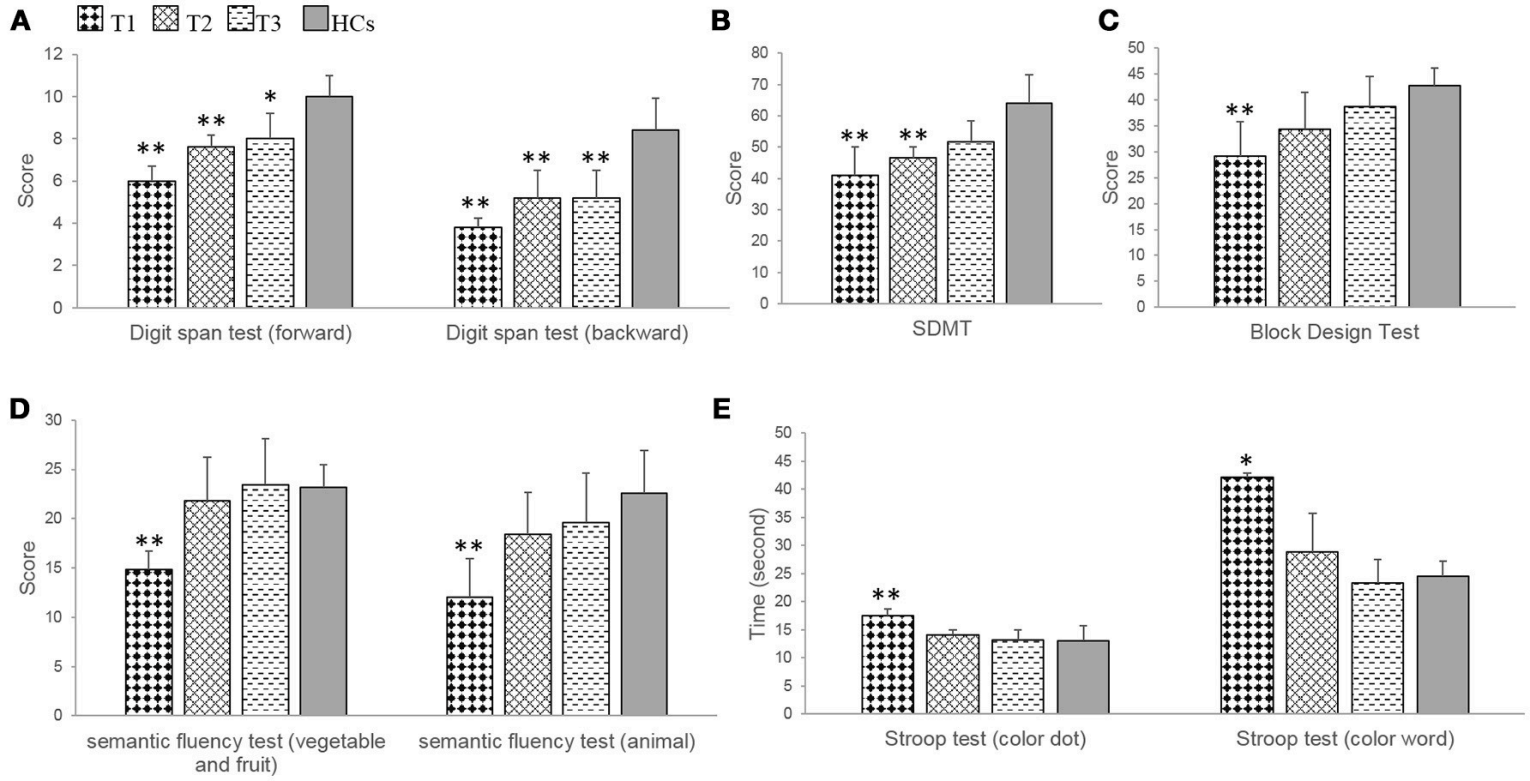
The results of neuropsychological tests related to frontoparietal cognitive function in the patients with anti-NMDAR encephalitis and health controls (HCs). **(A,B)** The patients showed demonstrated obvious improvements during the recovery period, but significant impairments were persisted at T1, T2 in digit span test and SDMT, and at T3 in digit span test. **(C–E)** The patients showed significant impairments at T1, and no obvious damage at T2, T3 in block design test, semantic fluency test and Stroop test. SDMT, symbol-digit modalities test. T1 = 1 to 2 months following initial immunotherapy. T2 = 6 months following initial immunotherapy. T3 = 11 to 12 months following initial immunotherapy. The two-sample *t*-test between the patients and health controls (HCs) at three time points. **p < 0.05/3; *p < 0.1/3 (Bonferroni correction).

In the digit span test, patients showed significant impairments at T1 (forward, *t* = −7.30, *p* = 0.000; backward, *t* = −6.51, *p* = 0.000), at T2 (forward, *t* = −4.71; *p* = 0.002; backward, *t* = −3.58, *p* = 0.007), and at T3 (backward, *t* = −3.58, *p* = 0.007) compared with HCs. Improvements in the forward subtest were demonstrated, and only marginally significantly impairments were found (*t* = −2.83; *p* = 0.022) (Table 3, Figure 3A).

Patients exhibited a significant improvement during the recovery period (forward, *F* = 21.00, *p* = 0.001; backward, *F* = 6.32, *p* = 0.023) (Table 3). Further multiple comparisons revealed significant improvements at all three time points in the digit span test (forward, T1 vs. T2, *p* = 0.009; T1 vs. T3, *p* = 0.010) (Table 4).

### Executive control

In the Stroop test, patients exhibited significant or marginally significant impairments at T1 compared with HCs (color dot, *t* = 3.40, *p* = 0.009; color-word, *t* = 3.57, *p* = 0.019). In contrast, at T2 and T3, significant improvements were observed and there were no significant differences between patients and HCs (Table 3, Figure 3B).

Patients showed a significant improvement during the recovery period (color dot, *F* = 24.12, *p* = 0.000; color-word, *F* = 8.07, *p* = 0.012). Further multiple comparisons showed significant improvements at all three time points in the Stroop test (color dot, T1 vs. T2, *p* = 0.001; T1 vs. T3, *p* = 0.009) (Table 4).

### Semantic fluency test

In the semantic fluency test, patients showed significant impairments at T1 (fruit and vegetables, *t* = −6.30, *p* = 0.000; animal, *t* = −4.01, *p* = 0.004) compared with HCs. In contrast, at T2 and T3, significant improvements were observed, and there were no significant differences between the patients and the HCs (Table 3, Figure 3C).

The patients showed a significant improvement in the semantic fluency test during the recovery period (fruit and vegetables, *F* = 12.05, *p* = 0.004; animals, *F* = 12.43, *p* = 0.004) (Table 3). Further multiple comparisons revealed a significant improvement at all three time points in the semantic fluency test (animal, T1 vs. T3, *p* = 0.041) (Table 4).

### Information processing speed

In the SDMT, patients showed significant impairments at T1 (*t* = −3.94, *p* = 0.004), at T2 (*t* = −3.94, *p* = 0.004) compared with HCs. In contrast, at T3, a significant improvement was observed, and there was no significant difference between patients and HCs (Table 3, Figure 3D).

Patients exhibited a significant improvement in SDMT during the recovery period (*F* = 5.45, *p* = 0.032). Further multiple comparisons revealed no significant improvements between T1 and T2, T1, and T3, T2 and T3 (Table 4).

### Visual-spatial capacity

In the block design test, patients exhibited a significant impairment at T1 (*t* = −4.07, *p* = 0.004) compared with HCs. In contrast, at T2 and T3, significant improvements were observed and there were no significant differences between patients and HCs (Table 3, Figure 3E).

Patients showed a significant improvement in the block design test during the recovery period (*F* = 13.38, *p* = 0.003) (Table 3). Further multiple comparisons revealed a significant improvement at all three time points in the block design test (T1 vs. T3, *p* = 0.017) (Table 4).

## Discussion

In the current study, we examined the recovery of adult patients with severe anti-NMDAR encephalitis. Following initial immunotherapy, a series of neuropsychological tests were conducted at three sequential time points. In the first stage (1–2 months following immunotherapy), comprehensive damage in cognitive function was present in all patients. Six months after immunotherapy, verbal episodic memory had substantially recovered. At approximately 1 year following treatment, there were no significant differences in the results of nonverbal episodic memory tests between the patient group and the normal control group. Nonetheless, working memory impairments remained in the patient group, mainly involving frontal functions. This neuropsychological evidence extends current understandings of the etiology and mechanisms of recovery of this disease.

Anti-NMDAR encephalitis is a diffuse encephalitis affecting the NMDA receptors that regulate synaptic transmission, thereby playing an important role in cognitive function (12, 13). This mechanism explains why comprehensive impairment of cognitive functioning is clinically present at an early stage. Our results are consistent with previous reports (6). Because the density of NMDA receptor distribution differs in different brain regions (usually highest in the hippocampus and second highest in frontal lobes), the clinical features of the patients are typically reflected in impairments of episodic memory and executive control (4, 5, 6).

Previous studies reported that episodic memory and executive function impairment were among the most common long-term sequela in anti-NMDAR encephalitis (2, 3). However, the results of the current study revealed a significant improvement in performance in the verbal episodic memory test and the Stroop color and word test during the second stage, approximately half a year after treatment, and in the nonverbal episodic memory test during the third stage, approximately 1 year after treatment. We speculate that there may be two reasons for the positive outcomes in episodic memory and executive function in patients with anti-NMDAR encephalitis. First, it was reported that early immunotherapy was uniformly and consistently considered as a favorable cognitive outcome indicator (2, 6). In the current study, all patients received immunotherapies at the early stage (within 3 months following the appearance of the first symptom). Second, the Stroop color and word test is an executive functioning measure of selective attention, cognitive flexibility, cognitive inhibition, and information processing speed (14, 15), which is insufficient for reflecting the full range of executive functions. It will be necessary for future studies to use more comprehensive tests of executive function, such as the Wisconsin card sorting test and delayed-response task, to explore the dynamic evolution of executive function in patients with anti-NMDAR encephalitis.

An interesting observation is the inconsistency in the speed of recovery between verbal episodic memory and nonverbal episodic memory. It took almost 1 year for the results of nonverbal episodic memory to recover. We believe this finding can be explained as follows. Time and scenario-dependent verbal episodic memory is closely related to the dominant mesial temporal lobe structures, whereas nonverbal episodic memory is related to non-dominant mesial temporal lobe structures, suggesting material-specific lateralization in episodic memory (16–21). Although there is robust evidence for the relationship between verbal episodic memory and left temporal structures, evidence of an association between right temporal lobe structures and nonverbal memory is not strong (22–24). During nonverbal episodic memory testing, it is difficult to avoid subjects' use of verbalization for nonverbal stimulus processing. If the subject applies certain speech-related tactics, it is possible that bilateral temporal function is involved during nonverbal episodic memory (21). Bilateral medial temporal lobe recruitment has been observed during nonverbal episodic memory encoding (17–19), indicating that nonverbal episodic memory may involve a more complex and extensive cerebral network (25).

It should be noted that, 1 year following immunotherapy, the patients showed no significant difference in almost all neuropsychological test results compared with the normal control group. Nonetheless, patients still exhibited a degree of impairment in the digit span test. It can be seen from the results that, compared with the normal control group, the patients' performance in this test had not reached the normal range, despite other significant improvements during recovery. We speculate that there are two potential explanations for this finding. First, following immunotherapy, adult patients with severe anti-NMDAR encephalitis may be left with mild impairments in working memory. Second, the timing of the third assessment of patients in this study was set at 1 year following immunotherapy; it is possible that cognitive function in these patients had not yet fully recovered. One previous study indicated that the recovery period for anti-NMDAR encephalitis may be as long as 18 months, or even longer (1). For these reasons, a longer follow-up period may be required to observe the full recovery of frontoparietal functions.

One of the limitations of the current study was the relatively small sample. This may have limited the statistical power for identifying some cognitive improvements that might have otherwise been detected early in follow-up examinations. In addition, because this study focused on patients with severe anti-NMDAR encephalitis, it remains unclear whether the same characteristics will apply to the recovery of cognitive function in mild or moderate cases. All patients received first-line and second-line immunotherapy at an early stage of the disease, it remains uncertain how much of an impact that on the patterns of cognitive recovery, because these therapies in themselves may have neuropsychological significance. Furthermore, although many neuropsychological tests were used, the examinations did not cover every aspect of cognitive function, and potential practice effects across time and ceiling effects of some of the neuropsychological tests should be considered. Future studies with larger samples, more comprehensive neuropsychological tests, and multimodal functional MRI examinations would be helpful for investigating the brain activation patterns in anti-NMDAR encephalitis. This could provide more direct evidence regarding the changes in cognitive mechanisms during the course of this disease.

## Ethics statement

This study was approved by the Ethics Committee of the First Affiliated Hospital School of Medicine, Zhejiang University. All patients and control subjects had signed the Informed Consent Form.

## Author contributions

ZC, KW, and BL designed research. ZC and DW performed experiments and analyzed data. ZC wrote the main manuscript text and prepared figures. KW and BL edited and revised manuscript. All authors reviewed and approved the manuscript.

### Conflict of interest statement

The authors declare that the research was conducted in the absence of any commercial or financial relationships that could be construed as a potential conflict of interest.
